# ALK-Activating Homologous Mutations in LTK Induce Cellular Transformation

**DOI:** 10.1371/journal.pone.0031733

**Published:** 2012-02-09

**Authors:** J. Devon Roll, Gary W. Reuther

**Affiliations:** Department of Molecular Oncology, Moffitt Cancer Center and Research Institute, Tampa, Florida, United States of America; II Università di Napoli, Italy

## Abstract

Leukocyte tyrosine kinase (LTK) is a receptor tyrosine kinase reported to be overexpressed in human leukemia. Though much regarding the function of LTK remains unknown, it shares a high degree of similarity with anaplastic lymphoma kinase (ALK), which is found mutated in human cancer. In order to determine if LTK has transforming potential, we created two LTK mutants, F568L and R669Q, that correspond to two well-characterized activating mutations of ALK (F1174L and R1275Q). LTK-F568L, but not wildtype LTK or LTK-R669Q, transformed hematopoietic cells to cytokine independence. LTK-F568L exhibited a stronger ability to induce loss of contact inhibition and anchorage-independent growth of epithelial cells compared to LTK-R669Q, while wildtype LTK was non-transforming in the same cells. Likewise, LTK-F568L induced greater neurite outgrowth of PC12 cells than R669Q, while wildtype LTK could not. Correlating with transforming activity, LTK-F568L displayed significantly enhanced tyrosine phosphorylation compared to wildtype LTK and LTK-R668Q and induced activation of various signaling proteins including Shc, ERK and the JAK/STAT pathway. Expression of wildtype LTK or LTK-R669Q generally led to weaker activation of signaling proteins than expression of LTK-F568L, or no activation at all. Thus, mutating LTK at residue F568, and to a lesser extent at R669, activates the receptor tyrosine kinase, inducing cell signaling that results in transforming properties. These studies suggest that aberrant activation of LTK may contribute to neoplastic cell growth.

## Introduction

Receptor tyrosine kinases (RTKs) are cell signal transducers which span the plasma membrane, binding ligands with their extracellular domain [1]. Ligand-binding typically triggers receptor dimerization, which in turn, causes the intracellular kinase domain to become activated. Subsequently, activation of an RTK's kinase domain leads to autophosphorylation and the phosphorylation of downstream targets that initiate signaling of various pathways in the cell. Leukocyte tyrosine kinase (LTK) is a RTK reported to be expressed in pre-B lymphocytes, B lymphocytes, and other hematopoietic cells, as well as brain and placenta [2]–[6]. It shares significant homology with fellow insulin-receptor superfamily member anaplastic lymphoma kinase (ALK) [7]. After the primary structure of LTK was partially determined in 1988 [2], Krolewski et al. reported full-length LTK to be a 100 kDa glycosylated protein with demonstrable *in vitro* kinase activity [8]. Although several splice variants of the protein exist, full-length LTK is generated from the predominate cDNA isoform that directs the synthesis of an 864 amino acid protein consisting of an extracellular domain, transmembrane domain, a tyrosine kinase domain, and a short carboxy terminus [4]. LTK contains two NPXY motifs (which mediate the recruitment of adaptor proteins for signal transduction) at Tyr485 and Tyr862, the former of which is highly conserved among members of the insulin receptor family [9]. Surprisingly, two decades after its cloning much remains unknown regarding this protein's function, largely because a ligand for LTK has not been identified yet.

While the specifics of mammalian LTK function are unclear, several studies have suggested that it plays an important role in growth and development. In mice, aberrantly activated LTK expressed from a transgene led to cardiac hypertrophy, cardiomyocyte degeneration, as well as gene reprogramming [10]. In zebrafish, LTK appears to be involved in fate specification of neural crest cells [11]. Furthermore, experiments conducted by Yamada et al. utilizing a chimeric LTK receptor suggest that LTK kinase activity promotes neurite outgrowth via PI3K/AKT and Ras/MAPK pathways [12]. Ueno et al.'s chimera work also demonstrated that human LTK can activate the Ras pathway, further implicating LTK in cell growth [9]. In pro-B cells expressing an EGFR/LTK chimera, LTK has been shown to associate with both IRS-1 (via Tyr485) and Shc (via Tyr862) and that both tyrosines contribute to activation of the RAS pathway and mitogenic signaling, while only Tyr485 contributes to anti-apoptotic signaling [13]. LTK associates with PI3K (via Tyr753), and this interaction is required for LTK to produce a survival signal in hematopoietic cells [14]. In addition, LTK has been reported to interact with other signaling proteins, including PLC-gamma and cRaf, in a LTK kinase-dependent manner [15]. Given LTK's ability to signal through both growth promoting and anti-apoptotic pathways, any dysregulation of the protein would be expected to carry important consequences for disease development, especially for neoplastic cell growth.

Maru et al. first reported a truncated form of human LTK, which was found to be expressed in 10 of 18 leukemia samples, including patient samples and cell lines, but not in 17 non-leukemic neoplastic cells examined [5]. This suggests a possible role for LTK in hematological malignancies. Further implicating LTK dysregulation in leukemia, the LTK gene was found to be overexpressed among 85 acute myeloid leukemia (AML) samples [16]. Subsequent studies by the same research group revealed that high expression of LTK in non-small cell lung cancer (NSCLC) patients correlated with a 3-fold risk of metastasis in stage I/II disease [17]. This suggests that LTK dysregulation may also have important consequences for cancer progression in this tumor type. Finally, Li et al. found that systemic lupus erythematosus (SLE)-prone mice harbor a gain-of-function polymorphism (Gly746Glu) in the LTK kinase domain near the PI3K binding motif [18]. The authors postulate that such a mutation may contribute to the aberrant activation of B cells seen in SLE. Taken together, just as LTK signaling studies imply, these findings also suggest that overexpressed and/or mutant LTK might contribute to disease. Clearly a better understanding of LTK is needed in order to ascertain its function in normal and disease states.

ALK is a protein highly related to LTK and together they are members of their own subfamily within the insulin receptor superfamily. Overall, the two proteins exhibit 54% identity of their overlapping regions. ALK is thought to play a role in normal nervous system development and function [7], [19]–[21]. Like LTK, ALK dysregulation has been implicated in carcinogenesis. However, more progress has been made in understanding the role of ALK in both normal and disease settings. One reason for this is that two possible ALK ligands (midkine and pleiotrophin) have been identified [22], [23], although whether they are truly ALK ligands remains controversial [24], [25]. In addition, various mutant forms of ALK have been reported in cancer. Full-length ALK was described in 1997 as a large glycosylated transmembrane RTK [7], [21]. The extracellular region of ALK is much larger than that of LTK and contains a number of domains that are not found in LTK—namely two MAM (merpin, A5 protein and receptor protein tyrosine phosphatase mu) domains and an LDLa (low-density lipoprotein class A) motif. While function of the LDLa motif in ALK is unknown, in the LDL receptor it is involved in ligand binding [26], [27]. MAM domains are thought to facilitate cell to cell interaction/adhesion [28] and the ligand binding domain for pleiotrophin and midkine fall within the first MAM domain [29]. However, both ALK and LTK share similar extracellular cysteine residues, glycine-rich domains in the extracellular region nearest the transmembrane domain, and NPXY motifs in their juxtamembrane regions [7], [28]. Importantly, the amino acid sequences of the ALK and LTK tyrosine kinase domains are nearly 80% identical.

A truncated form of ALK was first described as part of a transforming chimeric protein in non-Hodgkin's lymphoma, in which ALK sequences were found to be fused to sequences of the nucleophosmin gene product (NPM) [30]. Since that time, additional ALK fusion partners have been identified and transforming versions of ALK as well as aberrant ALK expression have been shown in cancers other than lymphoma including adenocarcinomas of the lung [31], [32], neuroblastomas [33]–[37], breast [38] and esophageal cancers [39], [40]. ALK is a frequent target of mutation in familial neuroblastoma, where alterations in the kinase domain lead to constitutive activation of the RTK and phosphorylation of downstream targets, resulting in heightened cell proliferation, invasion, and survival [34]–[37]. This is likely due to the ability of ALK to activate the Ras/ERK, JAK/STAT, and PI3K/AKT pathways. Among the ALK transforming mutations reported in neuroblastomas, mutations at kinase domain residues F1174 and R1275 are the most frequently reported [34], [35], and cells harboring these mutations have been shown to be sensitive to small molecule inhibitors of ALK *in vitro* [35], [41]. In fact, the ALK inhibitor crizotinib was recently approved for use in certain NSCLC patients [42], [43] and a number of other ALK inhibitors are currently in development or in clinical trials (reviewed in [44]). Additionally, ALK dysregulation has been found to carry histological and prognostic significance, underscoring the importance of these genetic changes in such cancers. For example, presence of the fusion protein EML4-ALK has been found to define histologically-distinct subsets of lung cancer [45], and ALK-positive anaplastic large cell lymphomas (ALCL) appear to have a better prognosis than ALK-negative ALCLs [46].

Though a substantial amount regarding the function of LTK remains unknown, including how it may become dysregulated in a disease state, the sequence similarity it shares with ALK may provide important clues. As mutations in the ALK kinase domain have been shown to be transforming, we hypothesized that this may be the case for LTK as well. Moreover, the ALK F1174 and R1275 mutational hotspots also correspond to known activating mutations in EGFR and ERBB2 [47]–[49], suggesting that such residues are key to regulating RTKs and thus likely LTK as well. In order to determine if LTK has similar transforming potential when mutated, we generated LTK proteins with mutations that correspond to these two most common activating mutations of ALK. Our goal in this study was to ascertain if altering these residues would result in gain-of-function signaling and transforming activity. Examination of the properties of such mutants is an important first step to better elucidating the possible mechanisms of LTK dysregulation in human malignancies. Our studies demonstrate that the activating ALK-homologous mutations in LTK differentially confer transforming activity on LTK.

## Results

### Generation and initial analyses of LTK F568L and R669Q mutations

**Figure 1:**
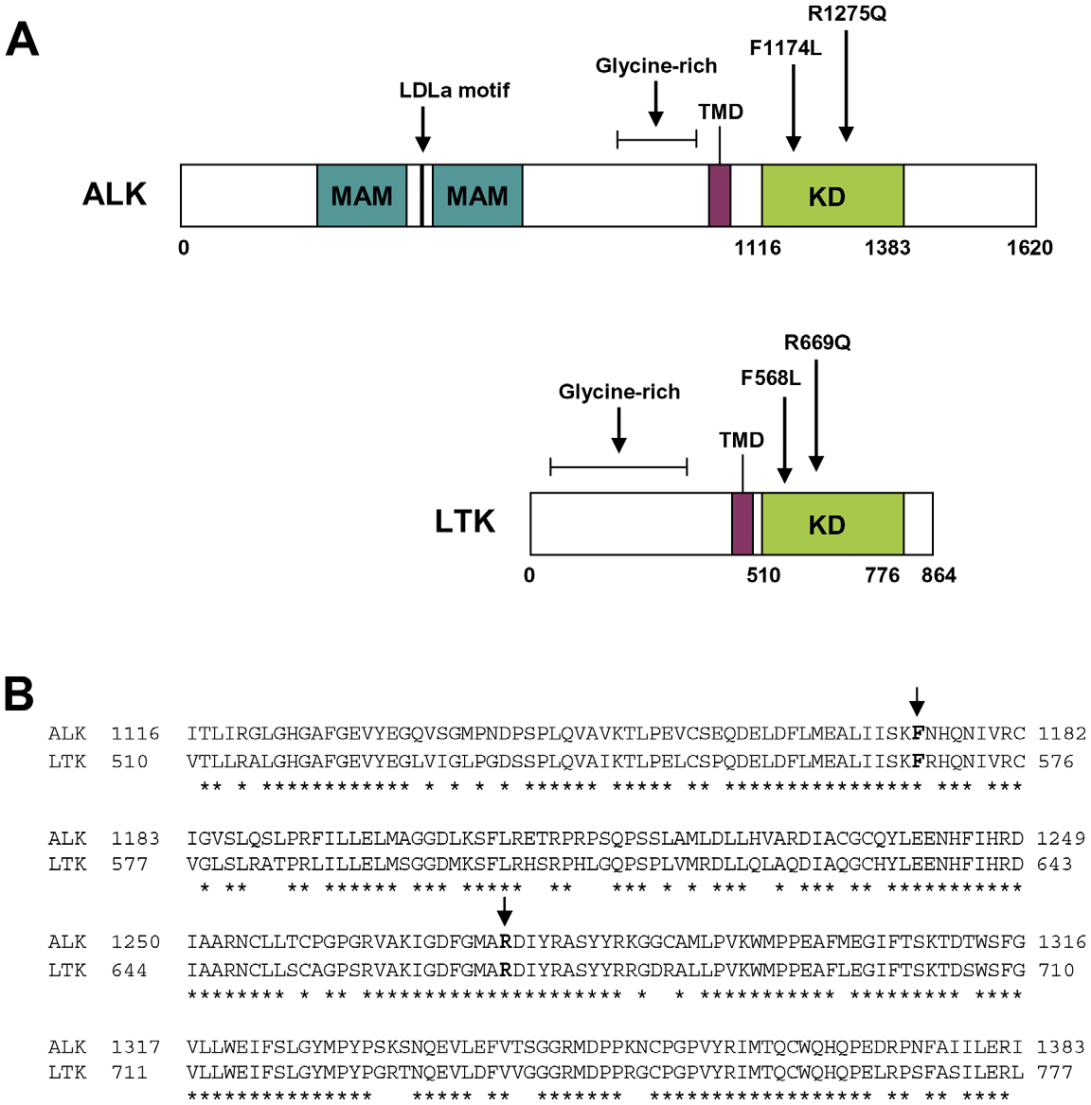
Comparison of ALK and LTK structure and sequence. (**A**) Schematic of ALK and LTK. ALK and LTK are RTKs, each containing an extracellular domain, a transmembrane domain (TMD, purple), and an intraceullar kinase domain (KD, green). ALK is a 1620 amino acid protein containing two MAM domains (blue) and an LDLa motif in its extracellular region. LTK is composed of 864 amino acids and has a much smaller extracellular domain that lacks these structures. The positions of the F1174L and R1275Q mutations of ALK and the corresponding mutations in LTK, F568L and R669Q, are indicated. (**B**) Alignment of ALK and LTK kinase domains. ALK and LTK proteins share 80% sequence identity in their kinase domains. Identical residues are indicated by an asterisk (*). Position of the F→L and R→Q residues of interest are shown in bold designated by arrows. Boundaries of the kinase domains were determined using the Simple Modular Architecture Research Tool, http://smart.embl-heidelberg.de/and the alignment was done using BLAST.

**Figure 2:**
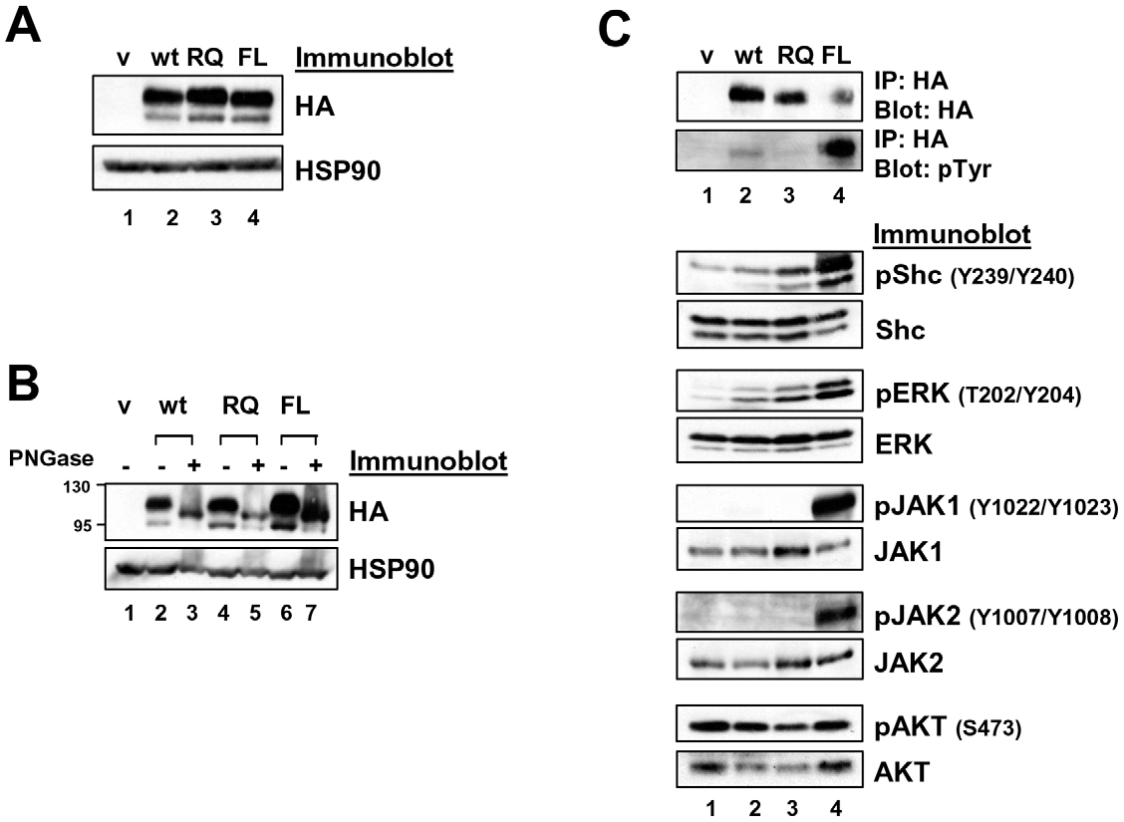
Expression of LTK constructs in 293T cells. (**A**) Relative protein expression of wildtype and mutant LTK constructs in 293T cells. Lysates of 293T cells transiently expressing either HA-tagged vector control (v, lane 1), wildtype LTK (wt, lane 2), LTK-R669Q (RQ, lane 3), or LTK-F568L (FL, lane 4) were immunoblotted with an anti-HA antibody as well as HSP90 as a loading control. (**B**) 293T cell lysates containing the LTK proteins were treated with PNGase F (lanes 3, 5, and 7) or left untreated (lanes 2, 4, and 6) and immunoblotted with HA antibodies and HSP90 as a loading control. (**C**) HA-tagged LTK proteins expressed in 293T cells were immunoprecipitated and immunoblotted with HA antibodies as well as anti-phosphotyrosine (pTyr) antibodies (top). Antibodies that recognize the indicated proteins (Shc, pShC-Y239/Y240, ERK, pERK-T202/Y204, JAK1, pJAK1-Y1022/Y1023, JAK2, pJAK2-Y1007/Y1008, AKT, and pAKT-S473) were used to immunoblot cell lysates of 293T cells expressing LTK proteins (bottom). The phosphorylated form of the protein is designated with a “p” preceding the protein name.

The ALK and LTK proteins are highly similar, sharing nearly 80% sequence identity in their kinase domains and 54% identity over their overlapping region (Figure 1). The ALK kinase domain mutations F1174L and R1275Q are two commonly reported activating mutations, particularly in familial neuroblastoma [34], [35]. In order to determine if mutations in the kinase domain of LTK possess a similar transforming potential as the known ALK mutations, we generated mutations at the F568 and R669 residues of LTK, which correspond to ALK F1174 and R1275, respectively (Figure 1B). We utilized a pBABE-puro HA-tagged retroviral expression vector to introduce mutant LTK into cells of interest. Expression of wildtype and mutant versions of LTK in transfected 293T cells revealed similar levels of expression for each HA-tagged LTK protein (Figure 2A). LTK protein migrated as a doublet, with the major form being approximately 115 kDa, a slightly larger molecular weight than has been reported previously [3]. We hypothesized that glycosylation, which has been reported previously in some species of LTK [8], may account for the observed size discrepancy. Therefore, we treated protein lysates from transfected 293T cells with PNGase F in order to remove protein glycosylation. Indeed, treatment with PNGase F resulted in a reduction in the size of the observed LTK protein, with the major band at ∼115 kDa shifting to an approximately 100 kDa band (Figure 2B), which is closer to the 92 kDa predicted molecular weight of the protein encoded by the cDNA that was expressed.

To determine if these LTK mutants induced activation of this RTK we analyzed expressed LTK proteins for tyrosine phosphorylation in transfected 293T cells. We examined tyrosine phosphorylation of LTK by immunoprecipitating HA-tagged LTK and immunoblotting for phosphotyrosine. Our analyses revealed that LTK-F568L demonstrated significantly enhanced tyrosine phosphorylation compared to wildtype LTK, while the LTK-R669Q did not exhibit elevated tyrosine phosphorylation (Figure 2C). We next examined various signaling proteins, some of which are known to signal downstream of LTK, for changes in phosphorylation status. Shc has been reported to be a downstream signaling target of LTK [9], and in fact, we detected a substantial increase in pShc (Y239/Y240) in the cells expressing LTK-F568L when compared to cells expressing wildtype LTK (Figure 2C). In contrast, cells expressing LTK-R669Q displayed only a slight increase in pShc relative to cells expressing wildtype LTK. Additional protein analysis of transfected 293T cells also revealed that expression of LTK-F568L led to an increase in pERK and a significant increase in pJAK1 and pJAK2 compared to expression of either wildtype LTK or LTK-R669Q (Figure 2C). Interestingly, expression of wildtype and LTK-R669Q did lead to elevated pERK compared to empty vector, but this activation was less than that observed with LTK-F568L. No enhancement of pAKT (Figure 2c), pSTAT3, or pSTAT5 (data not shown) was detected in this cell line.

### LTK-F568L transforms BaF3 and 32D cells to cytokine independence

**Figure 3:**
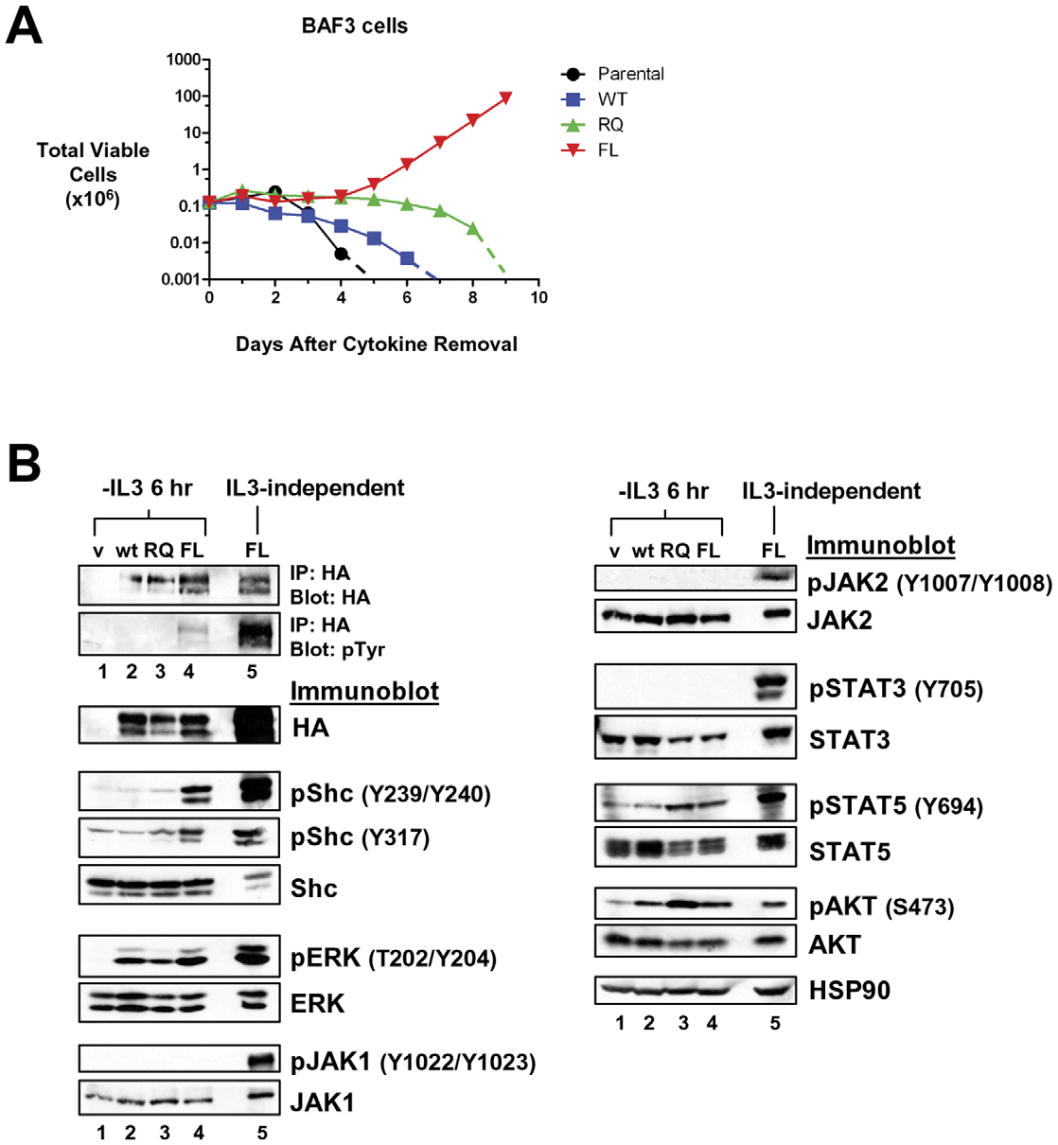
LTK-F568L transforms BaF3 cells to cytokine-independence. (**A**) BaF3 cells stably expressing LTK-F568L (red inverted triangles), LTK-R669Q (green triangles), or wildtype LTK (blue squares) and parental control cells (black circles) were cultured in the absence of IL-3 at Day 0. The total number of viable cells was determined at each timepoint by trypan blue exclusion. Similar results were obtained in three independent experiments. The dashed line indicates cell numbers going below the detection of the hemacytometer towards zero. (**B**) Lysates from cells stably expressing either HA-tagged vector control (v, lane 1), wildtype LTK (wt, lane 2), LTK-R669Q (RQ, lane 3), or LTK-F568L (FL, lane 4) were cultured in the absence of IL-3 for six hours and immunoprecipitated with HA antibodies, followed by immunoblotting with HA and pTyr antibodies (first two blots). The same lysates were immunoblotted with antibodies to the indicated cell signaling proteins (see Figure 2 legend plus pShc-Y317, STAT3, pSTAT3-Y705, STAT5 and pSTAT5-Y694). Cell lysate from IL-3-independent LTK-F568L-expressing BaF3 cells was also analyzed (lane 5).

**Figure 4:**
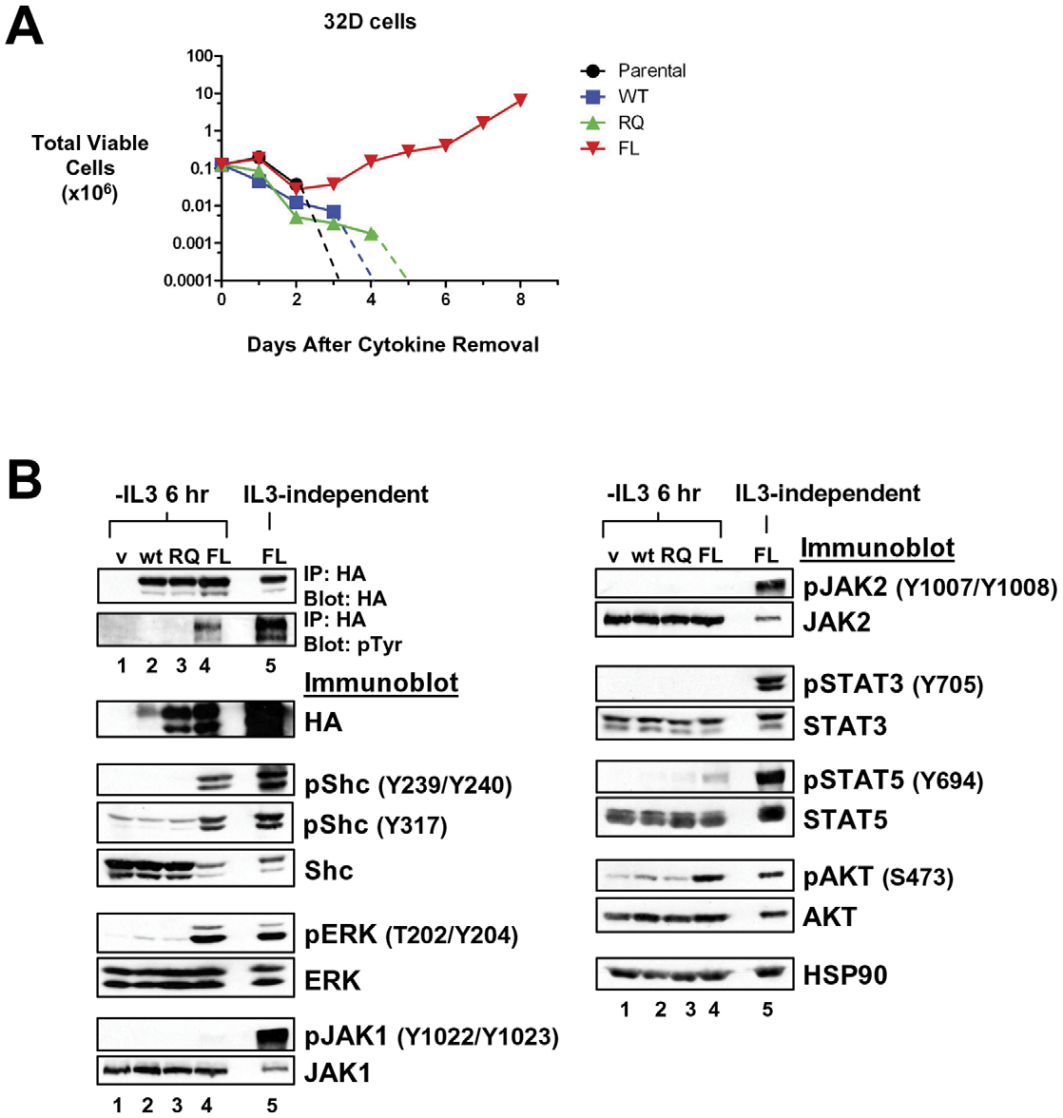
LTK-F568L transforms 32D cells to cytokine-independence. (**A**) 32D cells stably expressing LTK-F568L (red inverted triangles), LTK-R669Q (green triangles), or wildtype LTK (blue squares) and parental control cells (black circles) were cultured in the absence of IL-3 at Day 0. The total number of viable cells was determined at each timepoint by trypan blue exclusion. Similar results were obtained in three independent experiments. The dashed line indicates cell numbers going below the detection of the hemacytometer towards zero. (**B**) Lysates from cells stably expressing either HA-tagged vector control (v, lane 1), wildtype LTK (wt, lane 2), LTK-R669Q (RQ, lane 3), or LTK-F568L (FL, lane 4) were cultured in the absence of IL-3 for six hours and immunoprecipitated with HA antibodies, followed by immunoblotting with HA and pTyr antibodies (first two blots). The same lysates were immunoblotted with antibodies to the indicated cell signaling proteins (See Figure 3 legend). Cell lysate from IL-3-independent LTK-F568L-expressing 32D cells was also analyzed (lane 5).

BaF3 cells are a pro-B cell line and 32D cells are a myeloid progenitor cell line, both of which are dependent on IL-3 for viability and growth. These cell lines are used extensively to assess the transforming potential of oncogenes in a hematopoietic setting. ALK proteins containing either F1174L or R1275Q mutations are able to transform BaF3 cells to IL-3-independence [35]. To test if the F568L and R669Q mutants of LTK are capable of mediating the transformation of hematopoietic cells, we stably expressed wildtype, LTK-F568L, and LTK-R669Q in both BaF3 and 32D cells. When these cells were cultured in the absence of IL-3, cell viability and proliferation begin to decline within 24 hours. Cultures of either parental BaF3 cells or BaF3 cells expressing wildtype LTK become 100% non-viable within five to seven days, while wildtype LTK-expressing or parental 32D cells were all dead within three to five days (Figure 3A and 4A), as they are unable to proliferate in the absence of IL-3. In comparison, BaF3 and 32D cells harboring the F568L mutation became IL-3-independent (Figure 3A and 4A). BaF3 cells expressing LTK-F568L reach IL-3-independence about five to six days after IL-3 removal (Figure 3A), while the 32D cells expressing LTK-F568L became IL-3-independent approximately three to four days after cytokine removal (Figure 4A). However, the R669Q mutant of LTK did not transform BaF3 or 32D cells to IL-3-independence, as these cells responded to cytokine withdrawal in a manner similar to cells expressing wildtype LTK (Figure 3A and 4A). These data suggest that the F568L mutation has a higher transforming potential than the R669Q mutation in hematopoietic cells.

### LTK mutants induce activation of cell signaling in hematopoietic cells

We next investigated how expression of LTK proteins in hematopoietic cells affected activation of various signaling pathways. In order to eliminate signaling by IL-3, we cultured cells for six hours in the absence of IL-3 before immunoblot analysis. Similar to our results in 293T cells, LTK-F568L demonstrated enhanced tyrosine phosphorylation compared to wildtype LTK or LTK-R669Q in both BaF3 and 32D cells (Figure 3B and 4B, lane 4, first two panels). We analyzed for activation, via phosphorylation, various signaling proteins, including Shc (at both Y239/Y240 and Y317), ERK, AKT, JAK1, JAK2, STAT3, and STAT5. Comparing the data obtained from the two different hematopoietic cell lines, LTK-F568L expression lead to activation of Shc, ERK, STAT5, and AKT, while wildtype LTK or LTK-R669Q either did not activate these proteins or did not demonstrate consistent activation between the two cell lines (Figure 3B and 4B). Importantly, as Shc is believed to be a direct downstream target of LTK [13], it demonstrated high levels of phosphorylation at tyrosines 239/240 and 317 only in cells that expressed LTK-F568L. We also analyzed the phosphorylation state of signaling proteins after cells expressing LTK-F568L became IL-3 independent. The level of LTK-F568L protein increased dramatically in cytokine-independent transformed cells (Figure 3B and 4B, lane 5). This is likely due to a selective pressure resulting in optimization of signaling in the absence of IL-3, which provides a very potent anti-apoptotic as well as mitogenic signal. Not surprisingly, this correlated with a further increase in phosphorylation of Shc, ERK, and STAT5, and activation of JAK1, JAK2, and STAT3 was also now readily evident (Figure 3B and 4B, lane 5).

### LTK mutants require JAK activity to transform hematopoietic cells

**Figure 5:**
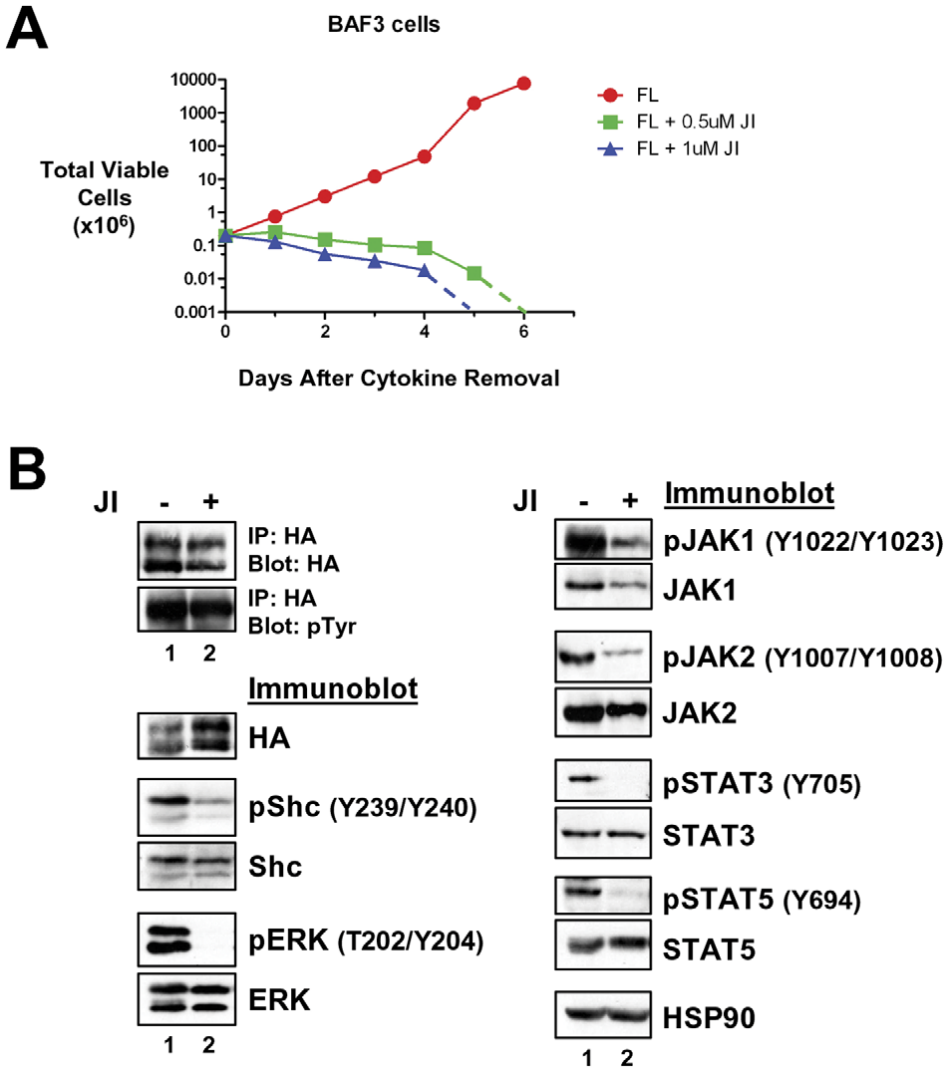
Transformation of BaF3 cells by LTK-F568L requires JAK family kinase activity. (**A**) BaF3 cells transformed to cytokine independence by LTK-F568L were cultured in the presence of 0.5 µM (green squares) or 1 µM (blue triangles) JAK Inhibitor I (JI) or 0.1% DMSO (red circles) on Day 0. The total number of viable cells was determined by trypan blue exclusion. Dashed lines indicate viable cell counts going below the limit of detection of the hemacytometer towards zero. Similar results were obtained in two independent experiments. (**B**) IL-3-independent LTK-F568L BaF3 cells were treated with 1 µM (lane 2) JI or DMSO (lane 1) for three hours and immunoblotted for the indicated proteins (See Figure 3 legend). Phosphotyrosine (pTyr) on LTK-F568L was detected by immunoblotting following immunoprecipitating LTK-F568L with HA antibodies.

The fact that cells transformed to IL-3 independence had significant activation of the JAK/STAT pathway following transformation to cytokine independence and not before, suggested this pathway may play an important role in cellular transformation in the context of LTK mutation in these cells. In order to assess the role of the JAK family kinases in the transformation of hematopoietic cell lines by LTK-F568L, we cultured LTK-F568L-transformed BaF3 cells with the pan-JAK inhibitor, JAK inhibitor I. JAK inhibitor I induced a dose-dependent decrease in cell viability (not shown) and growth in BaF3 cells transformed to cytokine independence by LTK-F568L (Figure 5A). As JAK inhibitor I is known to block phosphorylation of various STAT proteins and can prevent ERK1/2 activation downstream of JAKs, we examined the changes in the phosphorylation states of these proteins in BAF3 cells treated with the JAK inhibitor (Figure 5B). This analysis revealed a marked reduction in phosphorylated JAK1, JAK2, and STAT5, a dramatic loss of phosphorylated ERK and STAT3, a surprising reduction in Shc phosphorylation, yet no change in tyrosine phosphorylation of LTK-F568L (Figure 5B).

### Treatment of LTK-F568L Mutants with ALK Inhibitor PF-2341066

**Figure 6:**
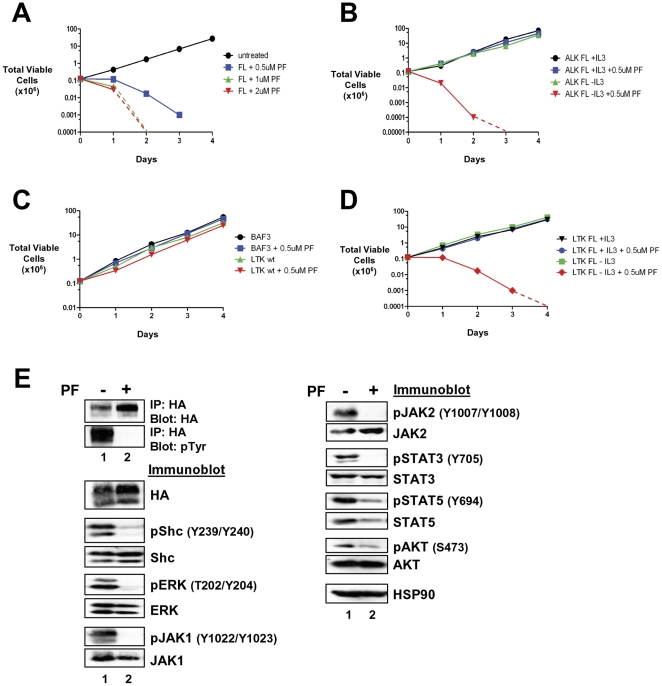
Transformed LTK-F568L BaF3 cells are sensitive to the ALK inhibitor PF-2341066. (**A**) BaF3 cells transformed to cytokine independence by LTK-F568L were left untreated (0.1% DMSO, black circles) or cultured in the presence of 0.5 µM (blue squares), 1 µM (green triangles), or 2 µM (red inverted triangles) of the cMET/ALK inhibitor PF2341066 (PF) on Day 0. The total number of viable cells for each treatment was determined daily by trypan blue exclusion. (**B**) IL-3-dependent (+IL-3) BaF3 cells harboring ALK-F1174L were cultured without (black circles) or with (blue squares) 0.5 µM PF2341066. BaF3 cells transformed to IL-3-independence by ALK-F1174L were cultured without (green triangles) or with (red inverted triangles) 0.5 µM PF2341066. The total number of viable cells for each treatment was determined daily by trypan blue exclusion. (**C**) Parental BaF3 cells were cultured without (black circles) or with (blue squares) 0.5 µM PF2341066. BaF3 cells expressing wildtype LTK were cultured without (green triangles) or with (red inverted triangles) 0.5 µM PF2341066. The total number of viable cells for each treatment was determined daily by trypan blue exclusion. (**D**) IL-3-dependent BaF3 cells expressing LTK-F568L were cultured without (black inverted triangles) or with (blue circles) 0.5 µM PF2341066. BaF3 cells transformed to IL-3 independence by LTK-F568L were cultured without (green squares) or with (red diamonds) 0.5 µM PF2341066. The total number of viable cells for each treatment was determined daily by trypan blue exclusion. In (A) thru (D), the dashed lines indicate viable cell counts going below the limit of detection of the hemacytometer towards zero. For each of the experiments in (A) thru (D), similar results were obtained in each independent (at least two) experiment performed. (**E**) IL-3-independent BaF3 LTK-F568L-expressing cells were treated with 1 µM PF-2341066 (lane 2) or DMSO (lane 1) for 2 hours. Cell lysates were collected and immunoprecipitated and/or immunoblotted, as indicated (see Figure 3 legend).

In order to determine if the sequence similarities between ALK and LTK could be exploited to target F568L-driven constitutive activation of LTK, we cultured BaF3 cells transformed by LTK-F568L with the cMET/ALK inhibitor PF-2341066 (Crizotinib). In the presence of this inhibitor, cell viability decreased (not shown) and cell proliferation was inhibited in a dose-dependent manner (Figure 6A). As a control we treated BaF3 cells transformed to cytokine independence by ALK-F1174L, with PF-2341066 and observed the expected inhibition of growth, only when the cells were dependent on ALK for growth (i.e. no IL-3 in medium) (Figure 6B). In contrast, when parental BAF3, wildtype LTK, or non-transformed LTK-F568L-expressing cells (all of which are dependent on IL-3) were treated with the inhibitor, growth and viability were unaffected (Figures 6C and D), suggesting PF-2341066 is not non-specifically toxic to these cells. PF-2341066 treatment abolished tyrosine phosphorylation of LTK-F568L (Figure 6E). We then examined the changes in the phosphorylation status of signaling proteins in response to PF-2341066 and found a marked reduction in the phosphorylation of Shc, STAT5, and AKT proteins and a complete disappearance of phosphorylated ERK, JAK1, JAK2, STAT3 proteins (Figure 6E).

### Transformation of epithelial cells by LTK mutants

**Figure 7:**
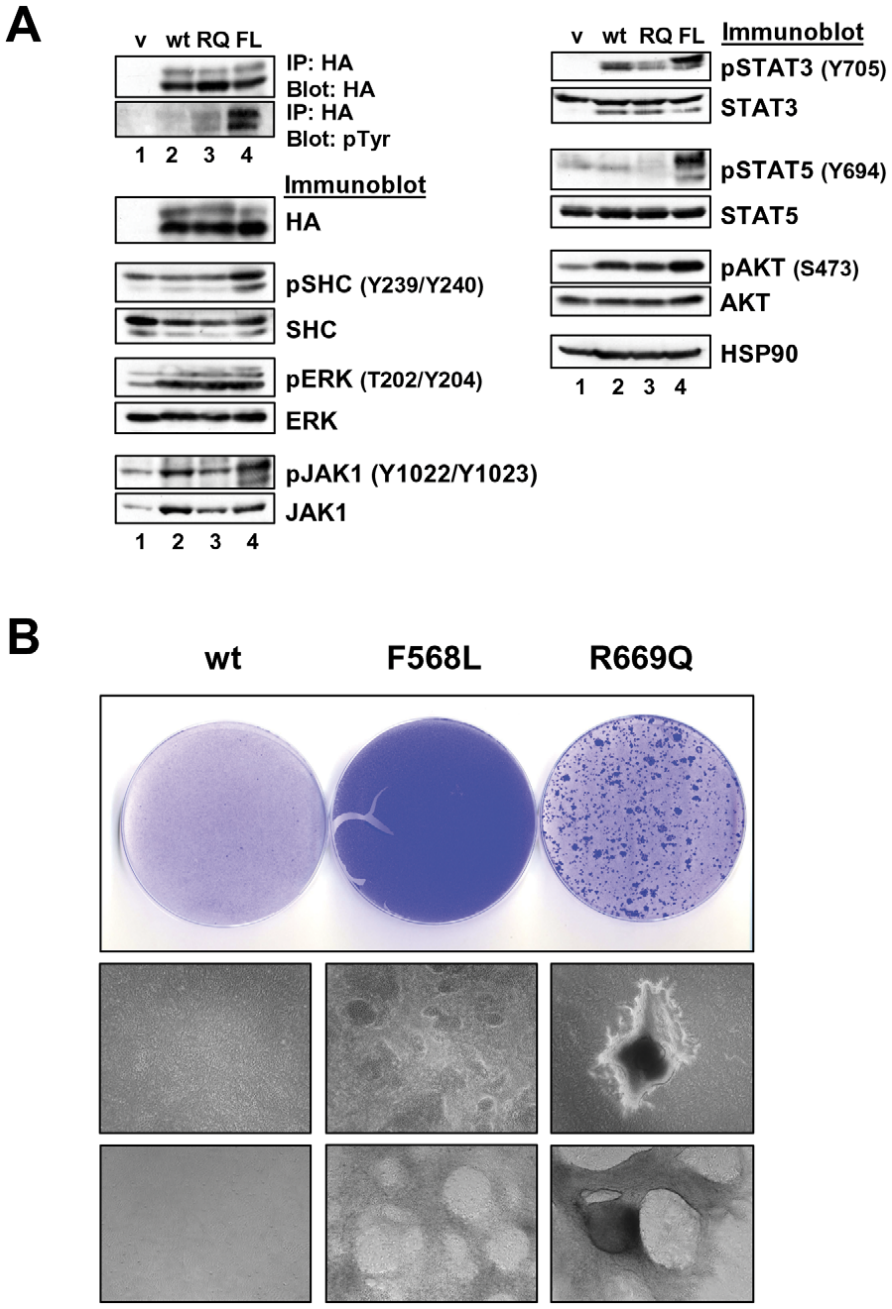
LTK-F568L and LTK-R669Q transform epithelial cells. (**A**) Cell lysates from RIE cells stably expressing either empty vector (v, lane 1), wildtype LTK (wt, lane 2), LTK-R669Q (RQ, lane 3), or LTK-F568L (FL, lane 4) were analyzed by immunoprecipitation and immunoblotting, as indicated (see Figure 3 legend). (**B**) RIE cells stably expressing either wildtype LTK (wt), LTK-F568L (F568L), or LTK-R669Q (R669Q) were plated and reached confluency after six days. Cells were cultured further and growth was grossly assessed for the loss of contact inhibition. Cells expressing wildtype LTK did not grow beyond confluency, continuing to exhibit contact inhibition. However, cells expressing LTK-F568L continued to proliferate, forming swirling patterns of cells growing on top of the monolayer. Cells expressing LTK-R669Q formed distinct secondary morphological structures as these cells formed compact clusters of cells that grew on top of the monolayer of cells. Representative photographs of cells are shown: plates were stained with crystal violet (top row) after photographs were taken at 10× with a digital camera (middle) and 50× with a Zeiss Automated Fluorescent Microscope using AxioVision (bottom row). Results shown are representative of similar findings of three independent experiments.

We next tested the signaling and transforming potential of mutant LTK proteins in epithelial cells. We generated rat intestinal epithelial (RIE) cells stably expressing wildtype LTK, LTK-F568L, or LTK-R669Q. Similar expression was obtained for each version of LTK (Figure 7A). We first analyzed these RIE cells for changes in activation of signaling proteins in response to LTK expression. While LTK proteins were equally expressed LTK tyrosine phosphorylation was significantly enhanced in cells expressing the F568L mutant of LTK, and a slight increase in tyrosine phosphorylation was detectable on LTK-R669Q compared to wildtype LTK (Figure 7A). Similarly, cells expressing LTK-F568L also contained elevated levels of phosphorylated versions of Shc, JAK1, STAT3, STAT5, and AKT compared to cells expressing control vector, wildtype LTK, or LTK-R669Q (Figure 7A). Interestingly, however, expression of wildtype LTK and LTK-R669Q, in addition to LTK-F568L, upregulated the phosphorylation of ERK, JAK1, and AKT compared to cells expressing an empty vector control (Figure 7A).

In order to determine if the F568L and R669Q mutations of LTK are able to transform these cells, we cultured RIE cells and allowed them to become confluent. Each of the stable lines became confluent six days after plating. By Day 11, the LTK-F568L cells had acquired a unique swirling morphology throughout the entirety of the plate as cells grew on top of each other, indicating an ability to escape contact inhibition (Figure 7B, lower panels). Interestingly, by Day 20, transformed colonies appeared in the LTK-R669Q plates. This morphology was different than LTK-F568L cells, with the LTK-R669Q-expressing cells forming compact dense clusters of cells (Figure 7B, lower panels). Wildtype LTK-expressing cells exhibited no sign of escaping contact inhibition of growth. The ability of LTK-F568L to induce contact inhibition is perhaps more evident following crystal violet staining, where cultures of these cells exhibited dense staining throughout the plate, compared to the monolayer of cells expressing wildtype LTK and the distinct foci of the cells expressing LTK-R669Q (Figure 7B, top panel). These results suggest that LTK-F568L, and to a lesser extent LTK-R669Q, are able to confer the ability of cells to escape normal contact inhibitory growth controls.

**Figure 8:**
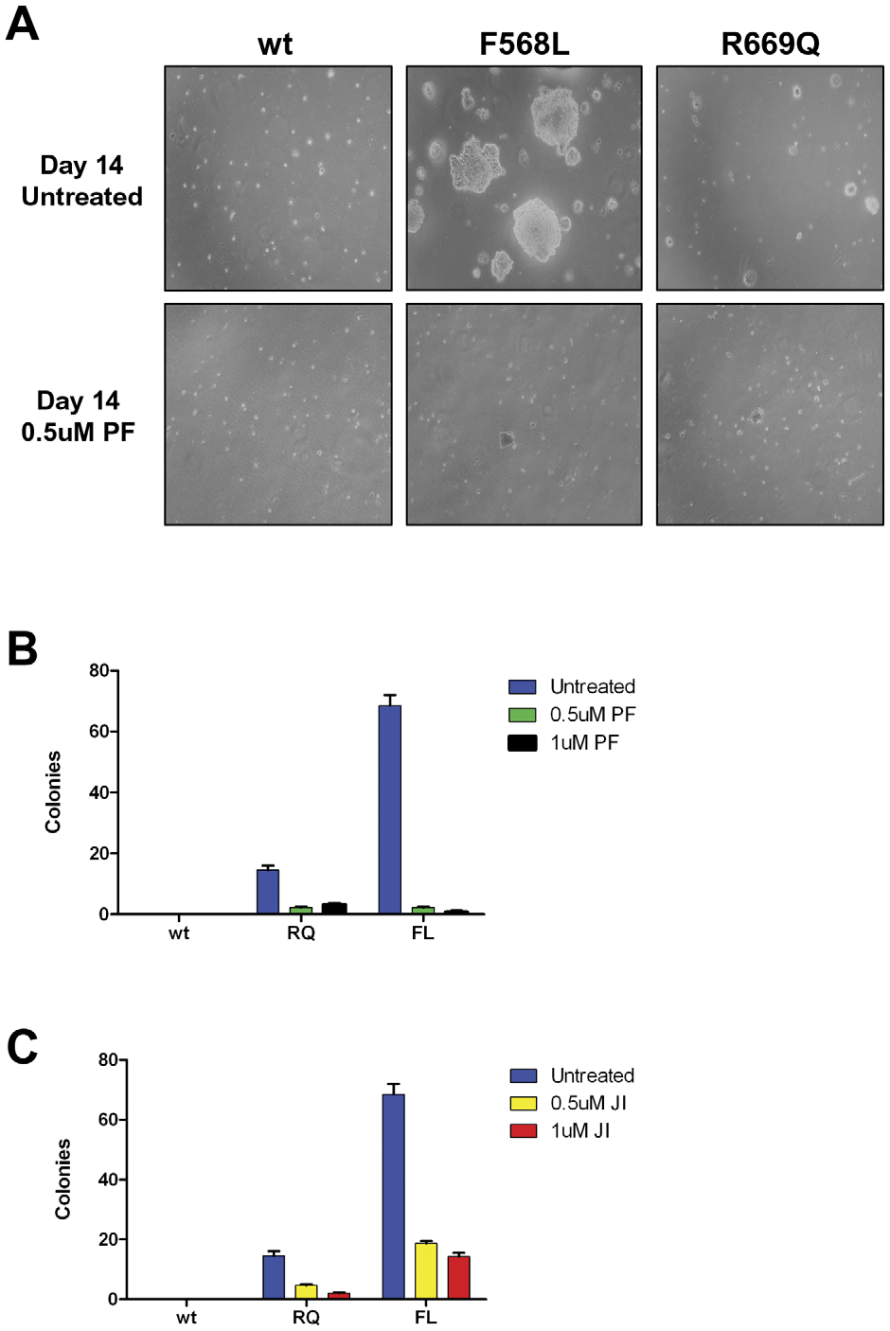
LTK-F568L and LTK-R669Q induce anchorage independent growth, which is inhibited by PF-2341066. (**A**) RIE cells stably expressing either wildtype LTK, LTK-F568L, or LTK-R669Q were plated in soft agar and assessed for anchorage-independent growth. LTK-F568L-expressing cells formed large colonies in soft agar while LTK-R669Q-expressing cells formed fewer colonies which were also significantly smaller. Including PF2341066 (PF) in the medium inhibited anchorage independent growth in soft agar. Photographs show representative fields after two weeks of culture. Results shown for are representative of similar findings of three independent experiments. (**B**) Quantitation of anchorage independent growth of RIE cells expressing wildtype LTK, LTK-F568L, and LTK-R669Q fourteen days after plating in the absence (blue bars) and presence of 0.5 µM (green bars) or 1.0 µM (black bars) PF2341066 (PF). (**C**) Quantitation of anchorage independent growth of RIE cells expressing wildtype LTK, LTK-F568L, and LTK-R669Q fourteen days after plating in the absence (blue bars) and presence of 0.5 µM (yellow bars) or 1 µM JAK Inhibitor (red bars). Data represents average colonies per field view per plate. Error bars represent standard error of the mean from two or three independent experiments performed in duplicate.

To further assess the transforming potential of LTK mutants, RIE cells containing either wildtype LTK, LTK-F568L, or LTK-R669Q were plated in soft agar to assess the ability of LTK mutants to induce anchorage independent growth. LTK-F568L and LTK-R669Q-expressing cells formed visible colonies five days after plating, while cells expressing wildtype LTK did not form colonies. Colonies of cells expressing the LTK-F568L mutant continued to grow in size, becoming much larger than the R669Q colonies by 14 days after plating (Figure 8A). Overall, LTK-F568L showed a stronger transforming phenotype than LTK-R669Q in this assay, forming six times more colonies than LTK-R669Q-expressing cells (Figure 8B). Thus, while cells expressing LTK-F568L readily formed colonies in soft agar, LTK-R669Q showed a weak transforming phenotype in this assay. While not as strong as LTK-F568L, the transforming ability of LTK-R669Q was still distinct from expression of wildtype LTK, which displayed no anchorage independent growth. Treatment with the ALK inhibitor PF-2341066 inhibited anchorage independent growth of both LTK-F568L and LTK-R669Q-expressing cells (Figure 8A–8B). A pan-JAK inhibitor also inhibited anchorage independent growth of cells expressing mutant LTK proteins (Figure 8C).

### Expression of LTK-F568L and LTK-R669Q mutants induce neurite outgrowth in PC12 cells

**Figure 9:**
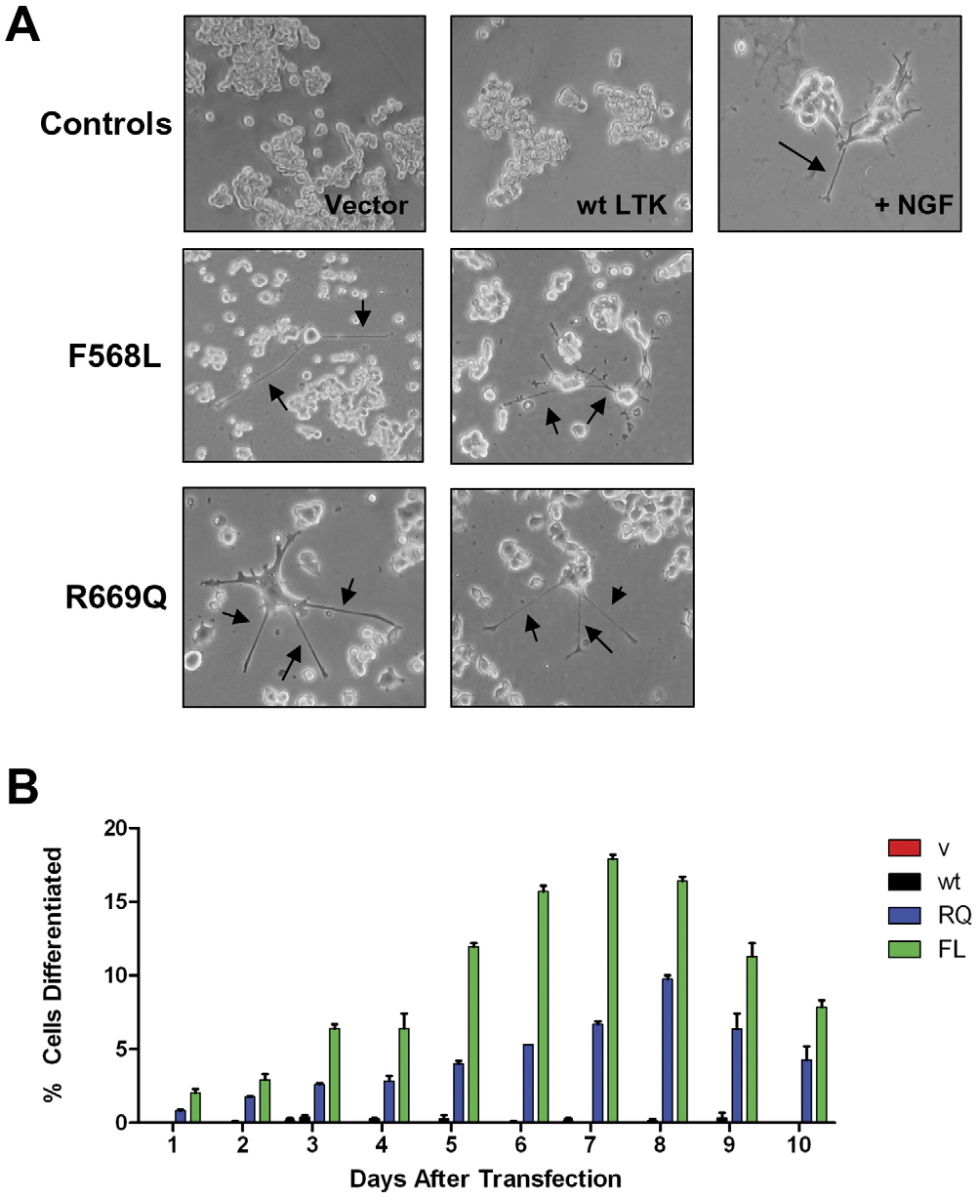
LTK-F568L and LTK-R669Q induce PC12 cell neurite outgrowth. (**A**) PC12 cells were co-transfected with GFP and either vector, wildtype LTK, LTK-R669Q, or LTK-F568L. Cells were observed for neurite outgrowth. PC12 cells treated with 1 uM NGF are shown as control for neurite extensions. Photographs of representative cells were taken four days after transfection. Arrows indicate neurite outgrowth. Two photographs for both LTK-F568L and LTK-R669Q are shown. (**B**) GFP-positive PC12 cells transiently expressing either vector control (v, red bars), wildtype LTK (wt, black bars), LTK-R669Q (RQ, blue bars), or LTK-F568L (FL, green bars) were counted daily for ten days and the percent exhibiting neurite outgrowth is shown. GFP was strongly expressed through Day 7, after which expression began to diminish and became undetectable after Day 10. Data shown is the average of two independent experiments done in duplicate with error bars representing standard error of mean. At least 500 GFP-positive cells were assessed per plate.

LTK has been reported to mediate neurite outgrowth when expressed as a chimera with CSF1R [12]. Upon stimulation with CSF1, such chimeras autophosphorylate CSF1R/LTK, leading to the formation of neurites from undifferentiated PC12 cells [12]. As a final analysis for deregulated LTK activity we expressed an empty vector control, wildtype LTK, LTK-F568L, or LTK-R669Q transiently in PC12 cells. LTK proteins were expressed with GFP and GFP-positive cells were assessed for differentiation and neurite outgrowth over a ten day period. Both LTK-F568L and LTK-R669Q were able to induce neurite outgrowth, as measured by the presence of cells with extended neurites longer than their bodies (Figure 9A). In contrast, vector-transfected cells as well as cells transfected with wildtype LTK did not differentiate (Figure 9A). When quantified, we found that 6.7% of GFP-positive LTK-F568L transfected cells and 2.7% of GFP-positive LTK-R669Q cells had neurite outgrowth by Day 3, while almost no (0.25%) wildtype LTK-expressing cells exhibited neurite outgrowth (Figure 9B). In comparison, when PC12 cells are treated with nerve growth factor (NGF), a strong inducer of differentiation, we observed that 26% of GFP-positive cells displayed neurite outgrowth by Day 3 (data not shown). We followed the GFP-positive cells for 10 days and found that the percentage of GFP-positive cells that exhibit neurite outgrowth peaked at Day 7, after which point the GFP signal began to fade. Seven days after transfection, 18.2% of GFP-positive LTK-F568L transfected cells and 6.9% of GFP-positive LTK-R669Q transfected cells exhibited neurite outgrowth, while no detectable neurite outgrowth was observed in wildtype LTK-expressing cells (Figure 9B).

## Discussion

Aberrant activation of various RTKs has long been associated with tumorigenesis. Point mutations in kinase domains of RTKs such as EGFR, HER2, MET, KIT, and FLT3 (among others) have been implicated as driver mutations in various cancers such as lung, breast, renal, liver, intestinal, and leukemia (reviewed in [50]). Such mutations tend to result in constitutive activation of the kinase domain, which ultimately contributes to escape from normal cellular growth controls. The gene for LTK, an RTK highly similar to ALK, is located within a chromosomal region implicated as a major breakpoint cluster domain in mouse models of radiation-induced AML [51]. Further evidence for the involvement of LTK in malignancies emerged when the gene was found to be overexpressed in a subset of AML patients [16] and overexpression of LTK was found to confer an increased risk of metastasis in NSCLCs [17]. Despite this knowledge, uncovering the specifics of LTK function has been hampered by the fact that the ligand(s) for this receptor is not known. Studies using chimeras constructed from the extracellular portion of the EGF receptor with the transmembrane and cytoplasmic domains of LTK have provided evidence that activation of the LTK kinase domain leads to signaling through the Ras pathway via Grb2 and the adaptor protein Shc [9]. Along with cell growth, LTK appears to be involved in anti-apoptotic signaling [13], [14]. Thus, disruption of normal LTK function may carry important consequences for neoplastic cell growth. For these reasons, we undertook the current study to investigate likely ways in which LTK could become constitutively activated and to observe the implications of such changes.

We utilized the high degree of conservation of kinase domain residues between LTK and ALK to inform our choice of candidate residues most likely to confer transforming properties when mutated. Two ALK residues in particular—F1174 and R1275—result in constitutive kinase domain activation when mutated in neuroblastomas [34], [35]. As with many kinase domain mutations implicated in cancer, the F1174 and R1275 mutations in ALK leads to phosphorylation of downstream targets and result in heightened cell proliferation, invasion, and survival [34]–[37]. We report here for the first time the consequences of the expression of LTK proteins containing mutations at the analogous sites to these ALK residues. Our analyses revealed that, in many ways, LTK-F568L and LTK-R669Q behave similarly to the F1174L and R1275Q mutants of ALK. Overall, the F568L mutation was a stronger activator of LTK signaling than the R669Q mutation (Figures 2, 3, 4, and 7). While R669Q mutant cells showed evidence of being able to escape normal growth controls (losing contact inhibition and exhibiting anchorage independent growth), this activity was considerably weaker than that of LTK-F568L (Figures 7 and 8). Additionally, while the F568L mutant of LTK was able to transform hematopoietic cells to IL-3 independence, LTK-R669Q was not (Figures 3 and 4). Such findings are consistent with research of the corresponding ALK mutations, wherein ALK-F1174L is considered more highly transforming than the R1275Q mutation [35].

The F568L mutation of LTK results in constitutive tyrosine phosphorylation of the receptor and expression of this LTK mutant leads to phosphorylation of several key signaling proteins that appear to act downstream of LTK (Figures 2, 3, 4, and 7). LTK has three phosphotyrosine sites that have previously been reported to be key in mitogenic and survival signaling: Y485, Y753, and Y862 [13], [14]. Tyrosine 753 of LTK is located within a kinase domain YXXM motif and appears to be involved in survival signaling via PI3K activation [14]. Tyrosine 485 of LTK is part of a NPXY motif located within the juxtamembrane domain which is highly conserved among the insulin receptor family (and which corresponds to Y1096 of ALK) [9]. Once phosphorylated, both Y485 and Y862 have been reported to associate with downstream signaling molecules, with Y862 being the major site of association with Shc resulting in the recruitment of Grb2/Sos and Ras activation [9], [14]. We found evidence of this LTK/Shc relationship, as numerous cell types expressing LTK-F568L revealed a marked increase in the phosphorylation of Shc tyrosines 239, 240, and 317 (known to be GRB2-binding sites) [52], [53], compared to cells expressing wildtype LTK (Figures 2, 3, 4, and 7). We also found evidence that activated LTK leads to phosphorylation of various proteins within the JAK/STAT pathway, including JAK1, JAK2, STAT3, and STAT5 (Figures 2, 3, 4, and 7), and that survival of hematopoietic cells transformed to cytokine independence by LTK-F568L expression requires JAK signaling. When hematopoietic cells transformed by LTK-F568L were treated with a pan JAK inhibitor, we found a decrease in or complete loss of the phosphorylated form of JAK1 and JAK2 as well as their downstream targets STAT3 and STAT5, as would be expected (Figure 5). Tyrosine phosphorylation of LTK remained unchanged during JAK inhibitor treatment. However, we observed a decrease in phosphorylated Shc and a complete disappearance of phosphorylated ERK in these cells. These data suggest, but do not prove, that activated JAK signaling contributes to Shc tyrosine phosphorylation and ERK activation downstream of activated LTK.

STAT3 activation and AKT phosphorylation have been reported following ALK-F1174L expression [34], [35]. Consistent with this, we also found evidence of STAT3 activation following the transformation of two hematopoietic cell lines by LTK-F568L as well as upon expression of this LTK mutant in epithelial cells (Figure 3, 4, and 7). When we examined mutant LTK cells for AKT activation, we found that in 32D cells only LTK-F568L expression increased AKT phosphorylation (Figure 4). In BAF3 cells the expression of LTK-F568L resulted in a slight increase in phosphorylated AKT (compared to vector control), while expression of LTK-R669Q exhibited a more marked increase in phosphorylated AKT in these cells (Figure 3). The opposite was true in epithelial cells, where LTK-F568L activated AKT to a greater extent than LTK-R669Q did (Figure 7). However, 293T cells failed to show any changes in AKT phosphorylation with expression of either mutation (Figure 2). Expression of ALK-R1275Q has been shown to lead to ERK1/2 activation [35], while results are conflicting as to whether ALK-F1174L does [34], [54] or does not [35] result in similar activation of ERK 1/2. In our experiments, we observed that LTK-F568L is as good and in some cell types a stronger activator of ERK than LTK-R669Q. Such findings suggest, not surprisingly, that cell type may play a role in determining which downstream signaling pathways become activated when a LTK mutation confers gain of function signaling activity.

In addition to holding important implications for hematopoietic cells, we found that mutant LTK confers important changes in cells of other types. In epithelial cells, both mutations were able to confer the ability to escape normal growth controls, including exhibiting anchorage independent growth (Figure 8). Additionally, our findings reveal that the F568L mutation of LTK is sufficient to induce differentiation of PC12 cells as measured by neuronal outgrowth (Figure 9). This provides additional evidence that LTK-F568L is a constitutively activated receptor tyrosine kinase. These observations are consistent with previous work utilizing a CSF1R/LTK chimera in PC12 cells, which suggests that LTK activation can signal through pathways resulting in neuronal differentiation [12]. Importantly, expression of LTK-R669Q also induced differentiation of PC12 cells, albeit to a significantly less extent than LTK-F568L (Figure 9). Nonetheless, this indicates LTK-R669Q is capable of inducing differentiation signals in PC12 cells, suggesting this mutant LTK does exhibit a level of increased signaling. In support of this, we observed that BAF3 cells expressing LTK-R669Q show an increase in phosphorylation of certain signaling proteins such as STAT5 and AKT, compared to wildtype LTK (Figure 3). Taken together, our data suggest that while LTK-R669Q does not readily exhibit potent transforming and cell signaling-inducing activity, expression of this LTK mutant does suggest it is a weakly activating mutation.

It remains to be determined whether or not activating LTK mutations are present in human cancer. Our work suggests that certain LTK mutations may have the ability to contribute to neoplastic cell growth, as has been demonstrated for ALK, whose kinase domain is nearly 80% identical to the kinase domain of LTK (Figure 1). Mutations of the corresponding residues of ALK have proved important in understanding the pathology of neuroblastomas that carry these genetic changes [34]–[37]. Moreover, the F1174 mutation of ALK occurs in a region of the kinase domain that is often mutated in EGFR and HER2 [55]. The R1275Q mutation of ALK is correspondingly adjacent to the most common lung cancer-associated mutation (L858R) in EGFR [55]. The similarity in the location of these ALK mutations, and thus the corresponding LTK mutations investigated in our study, to other activating tyrosine kinase domain mutations in cancer underscores the important consequences of mutation of this region of tyrosine kinases. Mutationally activated ALK is found in NSCLC and, interestingly, examination of LTK expression in patients with NSCLC revealed that patients with LTK overexpression had a three-fold higher risk of metastasis [17]. While our work shows that mutationally activated LTK can induce transformation of various cell types including epithelial cells, overexpression of wildtype LTK does not. However, overexpression of wildtype LTK does lead to activation of some downstream signaling proteins, such as ERK, in certain cell types (Figure 2, 3, and 7). Thus, it is possible that overexpression of LTK may contribute in some manner to enhanced signaling of distinct intracellular pathways, which if not significant on its own, may sensitize cells to additional genomic insults. Also, constitutively activated ALK is known to carry prognostic value in cancers such as lung cancer [45] and ALCL [46], [56], thus providing further evidence that mutations in LTK that induce constitutive signaling may provide clinically important information.

Importantly, we found that cells transformed by LTK-F568L are susceptible to the ALK inhibitor PF-2341066 (Crizotinib) (Figure 6 and 8). While little is known about the normal role of LTK, it is worth noting that treatment of cells with PF-2341066 to target mutationally-activated ALK may produce off-target effects through inhibition of LTK. Our work suggests that the similarities between ALK and LTK may be exploited for treatment options if LTK is found to have a role in driving certain cohorts of cancer patients. Having a potential therapeutic agent available makes the identification of potential activating LTK mutations in cancer all the more intriguing. While the size of the patient population with cancers containing activating LTK mutations, if any, is not yet known, advances in genomic sequencing, which will provide data for the personalization of therapeutic treatments for patients, makes the identification of such a population significant. This is especially true if these cancers can be effectively targeted by drugs already being used in patients. While further research is needed to elucidate the role of LTK in human cancer, the potential for improved prognosis is significant if LTK-driven neoplasms can be identified and met with targeted treatments. Future whole genome sequencing approaches will rely heavily on studies such as ours presented here to determine the significance of identified mutations.

In conclusion, we demonstrate that expression of LTK mutations homologous to known activating mutations of ALK leads to elevated activation and cell signaling compared to wildtype LTK. LTK-F568L is a stronger transforming mutation than LTK-R669Q in multiple cell types. Signaling and transforming activity of mutated LTK proteins are evident in cells of hematopoietic and epithelial origin, as well as in cells used to model neuronal differentiation, suggesting aberrant activation of LTK may play a role in neoplastic disease of multiple cell types.

## Materials and Methods

### Cell Culture and Retrovirus Production

293T [57] and RIE [58] cells were maintained in DMEM supplemented with 10% fetal bovine serum (FBS). BaF3 [57] and 32D [59] cells were grown in RPMI medium 1640 supplemented with 10% FBS and 5% WEHI-3B conditioned medium (as a source of IL-3). PC12 (ATCC: CRL-1721) cells were maintained in RPMI-1640 supplemented with 10% horse serum and 5% FBS. Ecotropic retrovirus was made in 293T cells using the pVPack system (Stratagene). Stable cell lines were generated by retroviral infection followed by selection with 1 µg/mL of puromycin as described previously [57].

### Construction of LTK Expression Plasmids

Wildtype LTK was amplified by PCR (PrimeSTAR DNA polymerase, Takara Bio, Inc.) from cDNA generated from reverse transcribed (Thermoscript Reverse Transcriptase, Invitrogen) mRNA from the leukemic cells of a patient with acute myeloid leukemia. The cDNA for LTK was cloned into pBabepuro-CHA [57]. The F568L and R669Q mutations of LTK were generated by PCR-mediated site-directed mutagenesis using PrimeSTAR DNA polymerase (Takara Bio, Inc.). Mutagenesis primers were as follows: 5′-CCC-TCA-TCA-TCA-GCA-AGT-TAC-GCC-ATC-AGA-ACA-T and 5′-ATG-TGA-TGG-CGT-ACC-TTG-CTG-ATG-ATG-AGG-G for the F568L mutation; and 5′-CTT-TGG-GAT-GGC-ACA-AGA-TAT-CTA-CCG-GG and 5′-CCC-GGT-AGA-TAT-CTT-GTG-CCA-TCC-CAA-AG for the R669Q mutation. The sequence of all cDNAs amplified by PCR was confirmed by DNA sequencing.

### PNGase Treatment

293T cells were lysed in lysis buffer (as described below, see Immunoblot Analysis) and protein concentration was determined by a BCA protein assay kit (Thermo Scientific). Fifty micrograms of protein were treated with PNGAse F (New England Biolabs), per manufacturer's instructions. Equal amounts of protein were analyzed by immunoblotting.

### Cell Growth Analysis

To assay 32D and BaF3 cell response to IL-3 deprivation, cells were washed twice with RPMI-1640 supplemented with 10% FBS. Cells were then plated at a concentration of 4×10^5^ per ml in RPMI-1640 supplemented with 10% FBS, and cell growth and viability were monitored over time by trypan blue exclusion.

### Immunoblot Analysis

Cells were washed in PBS and lysed in lysis buffer, composed of 25 mM Tris (pH 7.4), 150 mM NaCl, 25 mM NaF, 1% Triton X-100, 1 mM sodium vanadate, 2 mM sodium pyrophosphate, 10 µg/ml leupeptin, 2 µg/ml aprotinin, and 1 mM PMSF. Protein concentrations were determined with a BCA protein assay kit (Thermo Scientific), and equal amounts of protein were analyzed by SDS/PAGE. Primary antibodies used in this study include: phospho-(p)STAT5(Y694) (611964) (BD Biosciences); AKT (sc-8312), HSP90α/β (sc-7947), pJAK2(Y1007/Y1008) (sc-16566-R), Shc (sc-1695), STAT3 (sc-483), STAT5 (sc-835), ERK1/2 (sc-93) (Santa Cruz Biotechnology); HA (MMS-101R) (Covance); JAK1 (3344), JAK2 (3230), pAKT(S473) (4051), pERK(T202/Y204) (4370), pShc(Y239/240) (2434), pShc (Y317) (2431), and pSTAT3(Y705) (9138) (Cell Signaling); pJAK1(Y1022/Y1023) (44-422G) (Invitrogen); and ptyrosine 4G10 (05-321X) (Millipore). Primary antibodies were detected with corresponding horse radish peroxidase conjugated secondary antibodies (Thermo Scientific). Immunoblots were developed using ECL Western Blotting Substrate (Thermo Scientific).

### Immunoprecipitation

Approximately 8×10^6^ Baf3 and 32D cells were washed in PBS before being lysed in lysis buffer. Protein concentrations were determined with a BCA protein assay kit (Thermo Scientific). 500 µg of protein were combined with 10 µl HA-probe(Y-11) (sc-805) (Santa Cruz Biotechnology), 20 µl Protein A beads (Thermo Scientific), and brought to a final volume of 1 mL in lysis buffer. The solution was placed on a rotator overnight at 4°C. The immunoprecipitation reactions were spun down at max speed for 30 seconds at 4°C, and washed with 1 mL fresh lysis buffer. This wash was repeated three more times. The IP reactions were then resuspended in 25 µl sample buffer and boiled for 5 min at 95°C, before being analyzed by immunoblotting.

### JAK Inhibitor I Studies

BaF3 and 32D cells were plated at 2×10^5^ cells per ml in growth medium containing 0.1% DMSO, 0.5 µM, or 1 µM JAK inhibitor I (EMD Biosciences/Calbiochem). After addition of the inhibitor, cell growth and viability were determined over time by trypan blue exclusion. For soft agar assays, RIE cells were plated in soft agar with 0.5 µM or 1 µM of JAK inhibitor I.

### ALK Inhibitor Studies

BaF3 cells transformed by LTK-F568L were plated at 1.25×10^5^ cells per ml in RPMI-1640 supplemented with 10% FBS and either 0.1% DMSO, 0.5 µM, 1 µM, or 2 µM cMET/ALK inhibitor PF-2341066 (Crizotinib) (ChemieTek). After addition of the inhibitor, cell growth and viability were determined over time by trypan blue exclusion. For soft agar assays, RIE cells were plated in soft agar with 0.5 µM or 1 µM PF-2341066 (Crizotinib).

### Detection of Loss of Contact Inhibition

RIE cells stably expressing wildtype LTK, LTK-F568L, or LTK-R669Q were plated in 10 cm dishes at a density of 1×10^6^ cells per plate. Growth medium was replaced every other day during the entire experiment and cells were allowed to become confluent. Once confluency was reached, cells were monitored for the next three weeks for evidence of loss of contact inhibited cell growth and photographed. Plates were also fixed with 10% methanol/10% acetic acid and stained with 0.4% crystal violet for 3 minutes, before being rinsed with dH20, dried, and scanned.

### Soft Agar Assay

1×10^5^ RIE cells stably expressing wildtype LTK, LTK-F568L, or LTK- R669Q were plated in duplicate in DMEM/10% FBS containing 0.4% agar on top of DMEM/10% FBS containing 0.6% agar. In order to assess the relative number of colonies formed, the 60 cm plate was broken down into quadrants. A field view was selected at random within each quadrant, all colonies within that field view were counted, and the resulting counts averaged. For drug treatment during soft agar assays, cells were plated with 0.5 µM or 1 µM PF-2341066 (Crizotinib) or JAK inhibitor I.

### PC12 Cell Transfection

PC12 cells transiently co-expressing GFP and either wildtype LTK, LTK-F568L, or LTK- R669Q or vector control DNA were generated by nucleofection. Two million PC12 cells were resuspended in 100 µL Bio Ingenio Electroporation Solution (Mirus) along with 2 µg DNA of interest and 0.5 µg GFP expression plasmid. The suspension was transfected in duplicate according to the manufacturer's PC12 specific protocol (Lonza) and the suspension was transferred to 2 mL of RPMI 1640/10% horse serum/5% FBS and plated in 12-well dishes. The percentage of GFP-positive cells that exhibited neurite outgrowth was recorded each day for ten days.
